# Quantitative immuno-mass spectrometry imaging of skeletal muscle dystrophin

**DOI:** 10.1038/s41598-020-80495-8

**Published:** 2021-01-13

**Authors:** David P. Bishop, Mika T. Westerhausen, Florian Barthelemy, Thomas Lockwood, Nerida Cole, Elizabeth M. Gibbs, Rachelle H. Crosbie, Stanley F. Nelson, M. Carrie Miceli, Philip A. Doble, Jonathan Wanagat

**Affiliations:** 1 Atomic Medicine Initiative, Faculty of Science, University of Technology Sydney, Ultimo, NSW Australia; 2 Center for Duchenne Muscular Dystrophy, University of California, Los Angeles, Los Angeles, CA USA; 3 Department of Microbiology, Immunology, and Molecular Genetics, David Geffen School of Medicine and College of Letters and Sciences, University of California, Los Angeles, Los Angeles, CA USA; 4 ARC Training Centre in Biodevices, Faculty of Science, Engineering and Technology, Swinburne University of Technology, Hawthorn, VIC Australia; 5 Department of Integrative Biology and Physiology, University of California, Los Angeles, CA USA; 6 Molecular Biology Institute, University of California, Los Angeles, Los Angeles, CA USA; 7 Department of Neurology, David Geffen School of Medicine, University of California, Los Angeles, USA; 8 Department of Human Genetics, David Geffen School of Medicine, University of California, Los Angeles, Los Angeles, CA USA; 9 Department of Pathology and Laboratory Medicine, David Geffen School of Medicine, University of California, Los Angeles, Los Angeles, CA USA; 10 Veterans Administration Greater Los Angeles Healthcare System, Los Angeles, USA; 11 Division of Geriatrics, Department of Medicine, David Geffen School of Medicine at UCLA, 10945 Le Conte Avenue, Suite 2339, Los Angeles, CA 90095 USA

**Keywords:** Biophysical methods, Diagnostic markers

## Abstract

Emerging and promising therapeutic interventions for Duchenne muscular dystrophy (DMD) are confounded by the challenges of quantifying dystrophin. Current approaches have poor precision, require large amounts of tissue, and are difficult to standardize. This paper presents an immuno-mass spectrometry imaging method using gadolinium (Gd)-labeled anti-dystrophin antibodies and laser ablation-inductively coupled plasma-mass spectrometry to simultaneously quantify and localize dystrophin in muscle sections. Gd is quantified as a proxy for the relative expression of dystrophin and was validated in murine and human skeletal muscle sections following k-means clustering segmentation, before application to DMD patients with different gene mutations where dystrophin expression was measured up to 100 µg kg^−1^ Gd. These results demonstrate that immuno-mass spectrometry imaging is a viable approach for pre-clinical to clinical research in DMD. It rapidly quantified relative dystrophin in single tissue sections, efficiently used valuable patient resources, and may provide information on drug efficacy for clinical translation.

## Introduction

The dystrophin–glycoprotein complex (DGC) is a transmembrane protein complex that links the intracellular actin cytoskeleton to the extracellular matrix^1–5^ and confers structural stability to the sarcolemma during muscle contraction^4^. Loss of muscle cell adhesion due to genetic mutations in genes encoding the DGC components at the sarcolemma often result in muscular dystrophies. Duchenne (DMD) and Becker (BMD) muscular dystrophy are the most common forms and are characterized by an absence or decreased expression of dystrophin. DMD is a terminal illness caused by an X-linked genetic mutation and is usually diagnosed in boys at 2 to 3 years of age, whereas BMD tends to manifest later in life and has slower progression. Many muscular dystrophy therapeutic interventions aim to restore or partially restore dystrophin expression^6–8^. It is desirable to determine both the quantity and location of dystrophin in skeletal muscle when assessing the efficacy of therapeutics within clinical trials^9^ as various patterns of expression lead to differences in functional outcomes, regardless of the total amount of protein^10^. For example, low levels of homogenously distributed dystrophin in the sarcolemma provides greater protection against injury than higher levels sporadically distributed across individual muscle fibres^11^.

Therapeutic progress is hampered by the lack of consensus on appropriately sensitive and reproducible methods for quantification of dystrophin^12^. The difficulties of developing such methods include the absence of appropriate standards, frequent low expression, sporadic dystrophin-positive revertant fibers, and residual trace dystrophin^13^. Tissue heterogeneity of dystrophin may also obscure successful therapies, as DMD patients typically have less than 3% of normal levels^14^, and mice models show that 15% of normal expression is sufficient to provide significant benefits^11^.

The recent approvals of eteplirsen and golodirsen, the first two FDA-approved DMD-specific treatments, has exemplified the necessity for reproducible and sensitive dystrophin analyses^15,16^. The current standard in assessing dystrophin expression consists of concomitant Western blotting and immunofluorescence/immunohistochemistry (IF/IHC). Applications of these methods for quantification lack sensitivity and have poor reproducibility, especially when dystrophin expression is less than 25% of normal levels^17^. Inter-laboratory evaluations (n = 5) for the determination in BMD and DMD samples and healthy subjects report coefficients of variation of 23–223% for Western blots and 22–67% for IF/IHC^10^, which exceed the minimum recommendations of the FDA for bioanalytical assays^18^.

Alternate methods employ liquid chromatography-mass spectrometry (LC–MS). Canessa et al*.*^17^ standardized a parallel reaction monitoring method for the absolute quantification of dystrophin protein in as little as 25 µg of human muscle biopsies. The method required preparation of full‐length ^13^C6–Arg- and ^13^C6,15N2–Lys-labeled dystrophin with SILAC myotube lysates, prior to trypsin digestion and LC–MS analysis with sufficient sensitivity to measure ~ 1% of normal expression. Complete proteomic workflows on membrane-enriched fractions^19^ and whole muscle extracts^20^ from *mdx-4cv* mice and wildtype mice identified changes in the abundance of 197 proteins. Although these methods may prove useful for determination of efficacy of therapeutic intervention, they did not provide spatial location, and required complex sample preparation.

Emerging alternatives for the spatial quantification of proteins include immuno-mass spectrometry imaging (iMSI) and Imaging Mass Cytometry (IMC). Both techniques use laser ablation-inductively coupled plasma-mass spectrometry (LA-ICP-MS), and a metal-conjugated antibody for targeting proteins of interest^21^. iMSI encompasses generic laser ablation systems coupled to either quadrupole or time of flight mass spectrometers that are capable of measuring both low and high mass elements and has been applied to the determination of endogenous trace metals and various proteins of interest. For example, iron (Fe) and tyrosine hydroxylase were quantified and co-localized in two^22^ and three-dimensional images^23^ of the substantia nigra of murine brains to predict the risk of parkinsonian neurodegeneration. IMC utilizes the Fluidigm Hyperion instrument optimized for the detection of high mass elements and has been applied to the construction of single cell atlases and pathology landscapes of breast cancers^24,25^. Other applications include identification of protein expression in defined regions of interest^26^, where biases may be introduced. A number of standard algorithms are routinely applied to automate segmentation of regions of positive expression in IF imaging and molecular MSI, however their use in LA-ICP-MS bio-imaging and iMSI is limited.

Here we describe an iMSI method for sensitive and repeatable dystrophin quantification in mouse and human tissues that is suitable for assessment of efficacy of therapeutic interventions of DMD. We examined various image segmentation methods for identification of positive dystrophin expression. The method was developed and validated in murine and healthy human tissue, and the optimized protocols applied to four DMD samples of varying dystrophin expression.

## Results

### Validation of iMSI in wild-type and mdx murine and in human skeletal muscle

**Figure 1:**
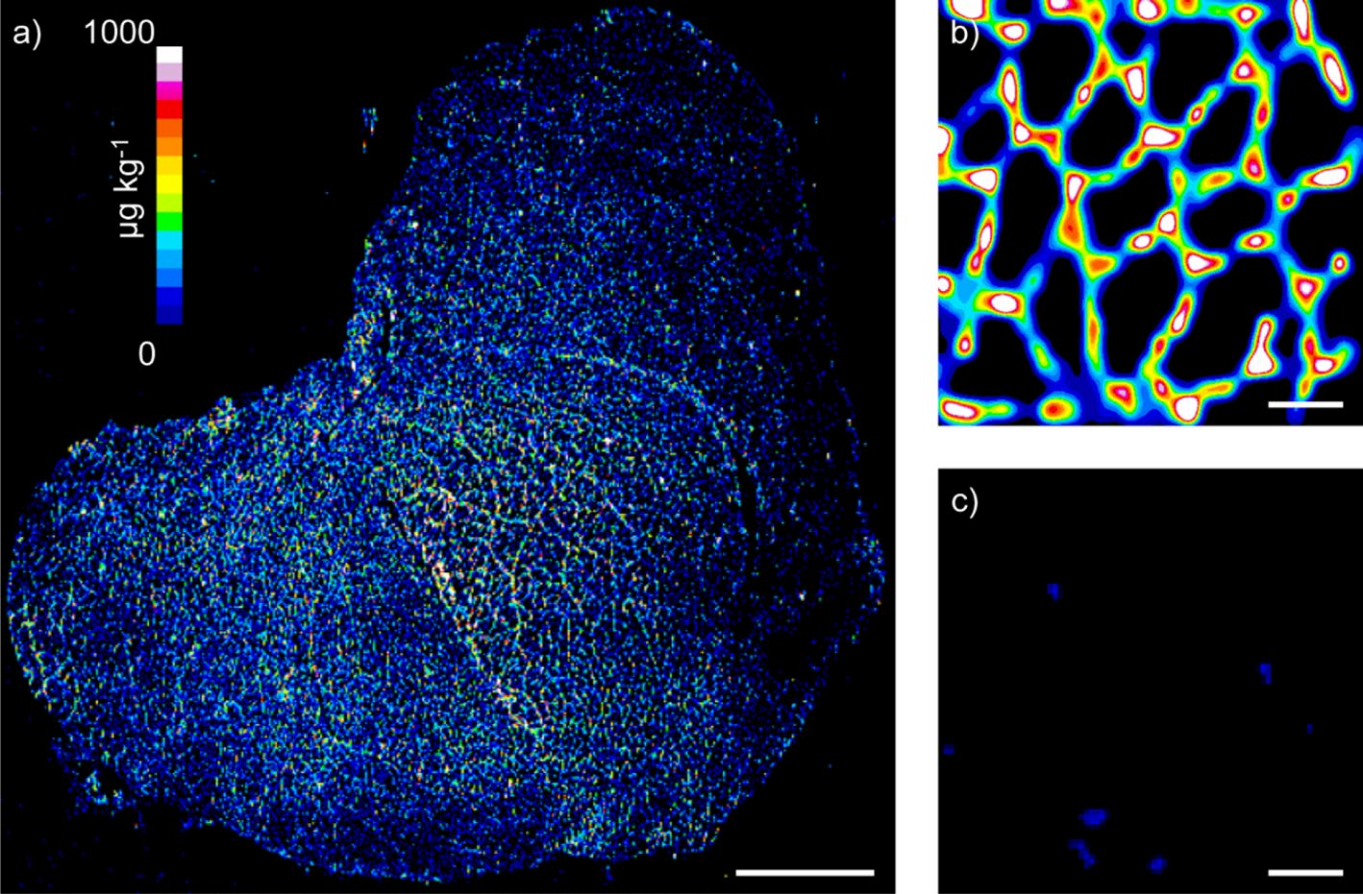
Dystrophin iMSI in wild type and *mdx* mouse quadriceps muscle. Low-resolution iMSI of whole wild-type mouse quadriceps cross section showing expected sarcolemmal distribution of dystrophin (**a**). High-resolution images of dystrophin iMSI in wild-type (**b**) and *mdx* mouse quadriceps (**c**). Quantification scale in a denotes µg kg^−1^ gadolinium for all panels. White bar denotes 1000 µm in (**a**) and 50 µm in (**b,c**).

The detection and spatial quantification of dystrophin was developed with Gd158-conjugated anti-dystrophin primary antibodies incubated in murine skeletal muscle tissue sections from wild type tissue and an *mdx* model used as a negative control of dystrophin expression. A full section image of dystrophin in a wild-type (WT) murine quadricep is shown in Fig. 1a, where the location and expression of dystrophin was determined by the Gd proxy, and quantified against external matrix-matched standards prepared in gelatine^27^. This low-resolution image of 125 µm^2^ per pixel clearly shows the expected sarcolemmal pattern, however, the resolution was inadequate to observe fiber-specific dystrophin. The resolution was increased to approximately 3.1 µm^2^ per pixel on a 300 × 300 µm region of interest of WT (Fig. 1b) and *mdx* mouse (Fig. 1c) quadriceps using super resolution reconstruction (SRR)^28^. The fiber-specific dystrophin distribution is clearly seen for the WT mouse, whereas little to no dystrophin expression was observed in the mdx mouse model with no revertant fibers identified within the region of interest.

**Figure 2:**
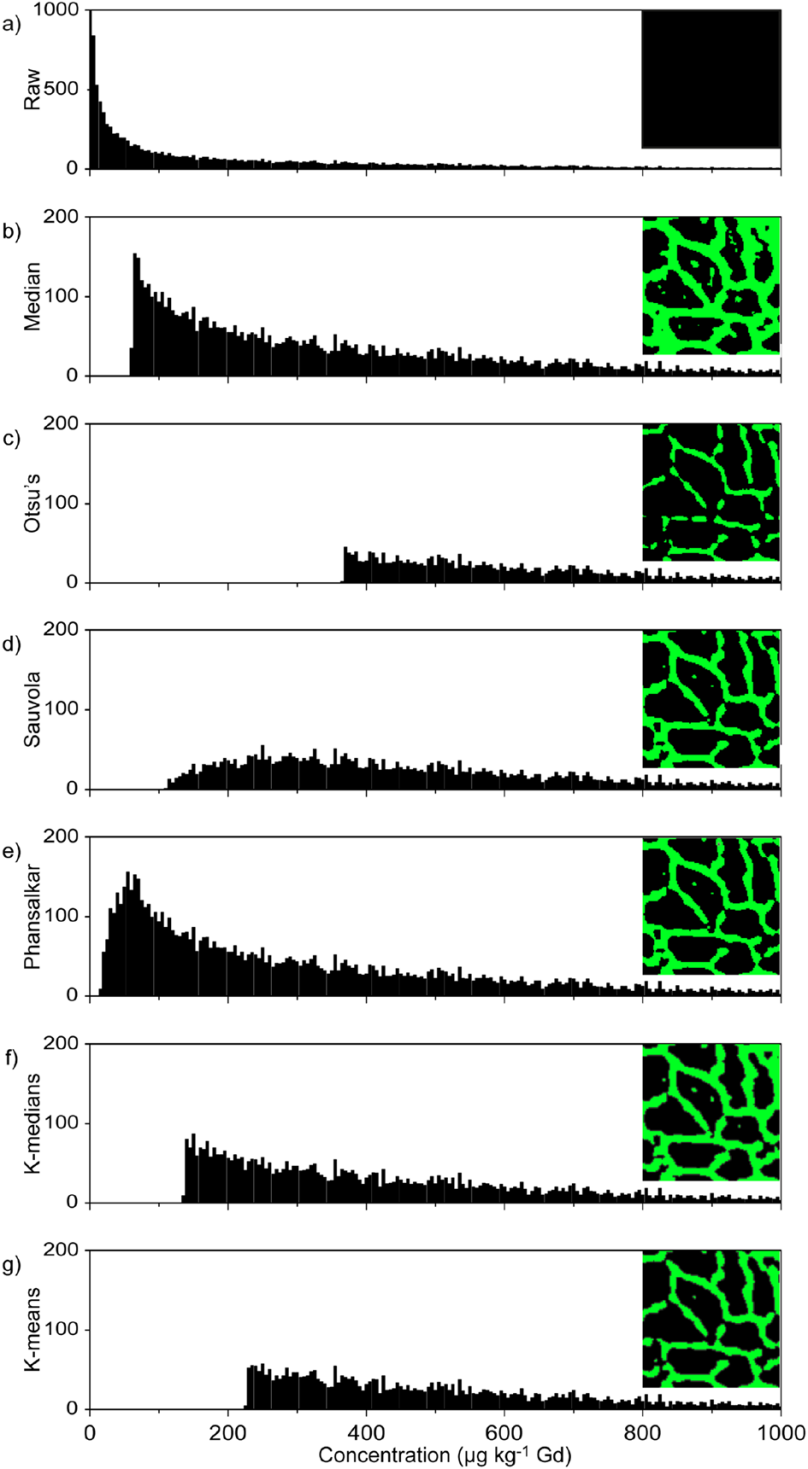
Histograms with the corresponding masks inset of the segmentation methods showing the data taken for quantification purposes using a representative sample image taken from Healthy human 1. The histograms contain the raw data (**a**), followed by the six different segmentation methods used, Median (**b**), Otsu’s (**c**), Sauvola (**d**), Phansalkar (**e**), k-medians clustering (**f**), and k-means clustering (**g**), respectively.

Post-acquisition segmentation algorithms were examined to objectively select dystrophin expression in the sarcolemma and eliminate null dystrophin areas located in the sarcoplasm (Fig. 2). These consisted of global, local, and k-clustering. Global approaches apply a threshold value calculated from across the entire image, local approaches apply a threshold calculated from the mean and standard deviation of neighbouring pixels^29^, and k-clustering segments data into “k” number of groups to minimize the Euclidean distances between the groups without supervision^30^.

Figure 2a shows the raw histogram of a 300 × 300 µm region of interest from a human quadricep section, Healthy 1. Quantification of the entirety of the image as represented by the corresponding mask (right inset) would bias the overall mean towards background signals and produce a value that was too low. The histograms and mask from calculation of two global thresholds, median and Otsu’s method^31^, are shown in Fig. 2b,c, respectively. The median mask clearly shows the characteristic sarcolemma pattern, with a bias towards lower values and therefore a thicker mask, whereas Otsu’s method biases higher values, and a thinner mask.

The histograms and masks from application of two local approaches of threshold calculation, Sauvola^32^ and Phansalkar^29^ methods, are shown in Fig. 2d,e, respectively. Both methods clearly segmented dystrophin in the sarcolemma, as well as null signal areas in the sarcoplasm. This observation was also apparent when applied to wild type murine 300 × 300 µm regions of interest (Supplementary Fig. S1a,b). Positive signals were also captured outside the sample in samples that contained large regions of background such as an image of a whole biopsy (Healthy 2, Supplementary Fig. S1c,d) due to minor differences in standard deviations or means in the local neighbourhood. Therefore, these local methods had a bias towards lower overall mean concentration.

The determination of segmentation thresholds using both k-means and k-medians failed to produce a consistent number of clusters with application of elbow and Bayesian inference criterion models. Instead, “k” values of 2 to 9 were heuristically applied, settling on a “k’ value of 3, which defined regions of higher expression that may be present in revertant fibers or stem cells in DMD sections without biasing the average value across the section. The histograms and corresponding masks for k-means and k-medians are shown in Fig. 2f,g respectively, with the concentrations obtained after averaging the second and third clusters. The expected sarcomeric distribution is clear in both images, however the median clusters behaved similarly to the local segmentation methods when imaging whole biopsies of low concentration DMD sections with background regions recognised as positive signal (Supplementary Fig. S1e). Supplementary Table S1 contains the concentrations and coefficients of variation (CV) obtained with the six segmentation algorithms for all samples analyzed. No one algorithm gave the highest concentration for all sample types, however k-means maintained an adequate CV, and Supplementary Fig. S1f shows that it was appropriate for the analysis of DMD sections. Therefore k-means clustering was chosen as the superior segmentation approach to provide the specificity and sensitivity to identify revertant fibers, and low abundant dystrophin expression presented by the majority of DMD cases^13^.

This optimized method was applied to the analysis of seven serial murine quadricep WT sections, and seven *mdx* sections. As before each acquisition consisted of a 300 × 300 µm region of interest within the tissue sections. External calibration was performed using gelatine standards before and after each acquisition, with the linearity of each run between 0.997 and 1.00. The lower limit of quantification of 69 μg kg^−1^ Gd (LLOQ) was calculated as 5 × the standard deviation of the blank. The average Gd concentration in WT murine quadriceps was 736 μg kg^−1^ Gd with an inter section CV of 10% (Table 1). The *mdx* mouse had an average Gd concentration of 85.0 μg kg^−1^.

**Table 1:** Gd concentrations obtained via iMSI as a proxy for dystrophin expression.

Sample source	Expected dystrophin expression	Gadolinium concentration^a^	CV
WT mouse	Normal	736^b,c^	10^b^
*mdx* mouse	Absent	85.0	20.5
Healthy 1	Normal	382^b,c^	19^b^
Healthy 2	Normal	286^b^	17^b^
DMD 1	Low	99.2	na^d^
DMD 2	Low	86.5	na^d^
DMD 3	Very low	< LLOQ	na^d^
DMD 4	Low	100	na^d^

*LLOQ* lower limit of quantification.

^a^µg kg^−1^ gadolinium.

^b^n = 7 consecutive muscle sections.

^c^Outlier removed after Grubb’s test (g_crit_ = 1.938 for n = 7).

^d^Single sections were analyzed from these samples so CV is not applicable (na).

### Determination of dystrophin in healthy subjects and DMD patients

Similarly, seven replicate serial sections from two healthy human quadriceps biopsies were stained using the same Gd158-conjugated anti-dystrophin primary antibody. The average concentration in Healthy subject 1 was 382 μg kg^−1^ Gd, and 286 μg kg^−1^ Gd in Healthy subject 2 (Table 1). Healthy 1 and Healthy 2 had similar homogeneity with CVs of 19% and 17% respectively.

**Figure 3:**
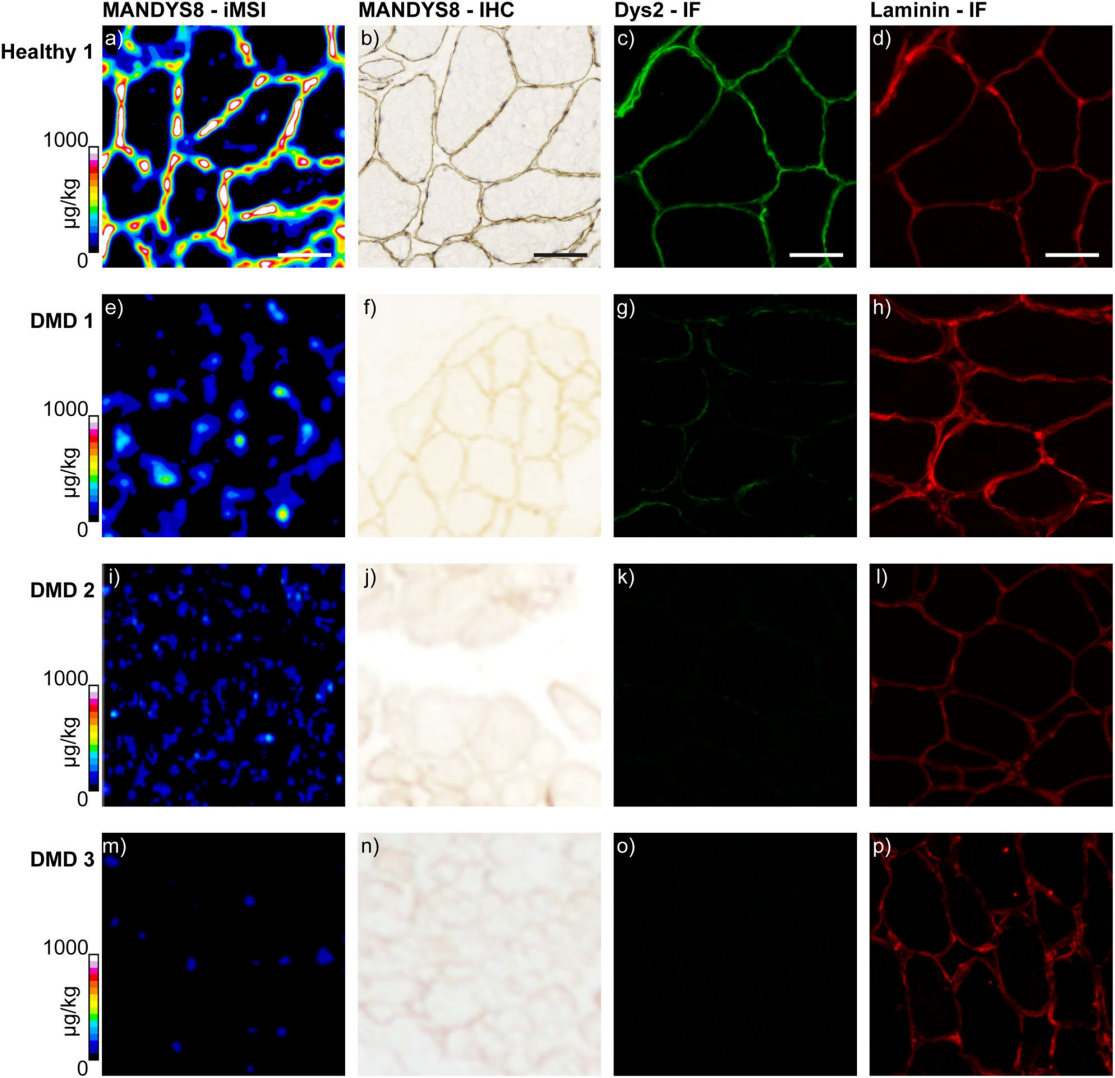
iMSI and histological localization of dystrophin in human muscle. iMSI for dystrophin using a Gd158-conjugated primary antibody (**a,e,i,m**). Dystrophin immunohistochemistry (**b,f,j,n**) and immunofluorescence (**c,g,k,o**). Laminin immunofluorescence (**d,h,l,p**). Healthy 1 (**a**–**d**); DMD 1 (**e**–**h**); DMD 2 (**i**–**l**); DMD 3 (**m**–**p**). Laminin immunofluorescence was used for muscle fiber localization. Quantification scales denote µg kg^−1^ gadolinium. Scale bars denote 100 µm in all images. All immunofluorescence images were taken using Axiovision 3.0 software.

Further serial sections of Healthy 1 were immunolabelled by standard methods for dystrophin IHC bright field or IF imaging. Laminin was also stained for IF imaging to highlight the structures of the sarcolemma. Figure 3a,b depicts the excellent spatial correlation between the iMSI and IHC images, with Figure 3c,d showing the expected sarcolemmal expression of dystrophin and laminin in healthy muscle.

Muscle samples were obtained from four DMD subjects that were expected to have differing dystrophin expression based upon genetic interrogations and clinical histories. Patient 1 had a mild case of DMD with an intronic mutation at exon 68 causing an out of frame 88 bp insertion. The patient was still ambulatory for short distances at the time of biopsy (16 yo), but lost ambulation at 18 years of age, which is later than usual for DMD (typically around 12 yo). Patient 2 was ambulatory for short distances at age 17 yo (age at the time of the biopsy) and had a nucleotide deletion at exon 30 (c.4100Del_A). Patient 1 and 2 had substantial variability of dystrophin expression with sporadic clusters of revertant fibers. These fibers bypassed the mutation allowing partial restoration of dystrophin expression. Patient 3 had a nonsense mutation at exon 70 leading to a premature STOP codon, therefore no protein was expected. This patient was ambulatory with stroller for long distance at the time of the biopsy (8 yo), with a loss of ambulation expected before 12 yo. Patient 4 had a nonsense mutation in exon 74 (c.10402G > T; E3468X), was still ambulatory at the age of biopsy (11 yo), and lost ambulation the following year at 12yo.

Muscle biopsies from each patient were imaged in the same manner as Healthy 1, i.e. stained with the primary and secondary antibody for IHC bright field imaging. After the IHC images were captured with a slide scanner, the coverslips were removed, and the sections imaged using iMSI. Serial sections were stained to obtain the IF images of dystrophin. Laminin was again imaged to obtain the location of the sarcolemma. The resulting image panel is shown in Fig. 3e–p. Dystrophin expression was of low abundance in all 4 cases and was consistent with the genetic characterization and clinical histories of these subjects, and as expected in the majority of DMD cases^13^. The dystrophin IF image of DMD 1 had the strongest intensity, followed by DMD 2, and DMD 3 showing no observable dystrophin. DMD 4 had similar expression to DMD 1 (images not shown). A similar laminin intensity was seen in all four cases.

The dystrophin levels as measured by iMSI correlated to the dystrophin levels observed in the IF and IHC stained sections, with the highest concentrations for DMD 1 and 4, a lower concentration in DMD 2, and < LLOQ for DMD 3 (Table 1).

## Discussion

The large number of possible mutations to the dystrophin reading frame that results in reduced or no transcription confounds DMD clinical research. Up to 62% of subjects demonstrated fibers with residual or trace dystrophin^13^. Those with nonsense mutations are predicted to be “typical” DMD patients based on history and the age at loss of ambulation^33–36^. These subjects usually present with no or low dystrophin as their mutation results in the formation of a truncated transcript^37^, causing degradation of unstable mRNA and complete absence of the protein^38^. Phenotype heterogeneity that results in variable dystrophin expression within the DMD population is well characterized^36,39^, however the effect of small mutations such as indels are harder to predict^40,41^.

These differences highlight the difficulties with associating dystrophin expression with a clinical outcome, and the necessity for a sensitive, robust technique to measure dystrophin as a biomarker for therapeutic intervention^42^. Despite several drugs under development restoring dystrophin production in DMD patients, the lack of standardized methods provides conflicting evidence leading to doubt that dystrophin is an appropriate biomarker of therapeutic efficacy. For example, Straub et al. report that current methods have not clearly established correlations between dystrophin levels and muscle function^42^, whilst Muntoni et al. suggest that there is strong correlation of dystrophin levels from a muscle biopsy, and is a superior biomarker than genetic predictions^43^. In contrast, Godfrey et al. report muscle strength and dystrophin levels were proportional^11^.

The iMSI data shown here were collected over several days and quantified against external calibration curves obtained before each acquisition, which allowed a robust comparison between samples. The results shown in Figs. 1 and 3 provided simultaneous localization and quantification of dystrophin on single muscle sections. These sections are reliably obtained from muscle needle biopsies, and unlike Western blots, did not require bulk tissues obtained from more intrusive surgical procedures. The method is compatible with standard IHC/IF workflows with reduced complexity as a secondary antibody was not required. The high-resolution images were suitable to measure dystrophin in the range of target samples including low expression typically observed in DMD patients. Furthermore, the iMSI method was calibrated against easily prepared traceable and validated external standards to facilitate facile comparison between clinical studies and sites. Another advantage of iMSI is that unlike LC–MS where quantification is affected by changes in the matrix, ionization of the element in the plasma is independent of the sample. Therefore quantification and comparisons of concentrations across multiple samples will not be affected by slight changes in sample preparation or the addition of other reagents such as a secondary antibody or other primary antibodies. The protocol was validated for murine and human tissue and therefore may be applied throughout the stages of drug development and clinical trials.

Segmentation was essential to gain an accurate objective representation of dystrophin expression within each region of interest by removing background signals and reproducibly determining positive signals. Global segmentation methods apply binary calculations to ascertain positive or negative pixels, with all the pixels below a certain value removed from the calculation of the average concentration across the image. Median and Otsu’s thresholding methods are routinely applied to IHC investigations and are the foundation of more complex thresholding and segmentation algorithms^44^. The dystrophin concentration calculated with Otsu’s method was at a higher concentration and had fewer outliers than median thresholding due to the inherent bias of median thresholds towards signals which occurred more often in the sarcoplasm (see Supplementary Table S1). However, the lack of a clearly bimodal histogram required a trade off in the threshold value as it implied the background and the positive signal may be merged^45^.

Local segmentation methods examine the signal standard deviation at adjacent pixels to calculate localised thresholds. When large standard deviations were identified, the image was segmented into positive and background regions. Sauvola’s method performs well when there is a high contrast of standard deviations within an image, however the method fails where there are low contrast regions^29^. Phansalkar adapted the Sauvola method to identify low contrast and high contrast positive cells^29^. The large variance in Poisson flicker noise from the ICP-MS^46^ results in pixels which will be incorrectly identified as positive signal. This was observed in Supplementary Fig. S1c,d, where standard deviations in the noise in areas outside of the tissue differed between neighbouring pixels and were represented in the mask, reducing the average concentration obtained across the section (see Supplementary Table S1).

K-means clustering consists of an unsupervised algorithm to partition unlabeled data into “k” number of groups. K-means is equivalent to a multilevel Otsu’s method and is easily expanded to a higher dimensional data set^47^. The k-means algorithm begins with the selection of “k” random centers from which the clusters are built. However, this can result in non-optimal clustering and is not always repeatable, even with robust center initiation^48^. K-medians is a variation of k-means clustering where the selection criteria are based on medians. One disadvantage of using k-means and other multilevel approaches is the subjective nature of parameter selection. Small changes in values of “k” can greatly affect the outcome of the segmentation. While there are some techniques that are useful to determine appropriate values of k, there is always some level of ambiguity. For example, the elbow method requires manual selection and others that select “k” based on a scoring function such as the Bayesian inference criterion are prone to overestimation^49^.

Nevertheless, these segmentation algorithms overcome the limitations of typical iMSI or LA-ICP-MS imaging analyses which often rely on manual selection of regions of interest to determine location specific concentrations^22,26^. Previous use of k-means clustering segmented anatomical regions of tissues, however required multiple elements to obtain clustering^50,51^. Similarly, other approaches also require multiple markers and consist of user-intensive segmentation based on fuzzy cluster analysis to show endogenous metals in neuroanatomical structures^23^, and segmentation of neighboring cells with multiple membrane markers^52^. Recent improvements of dystrophin measurement to identify dystrophin positive fibers use a second membrane protein as a mask. For example, spectrin has been used as a control protein to normalize dystrophin IF images in DMD, BMD, and wild-type muscle fibers with no statistical difference between samples stained and imaged over two separate days^53^. Beekman et al. developed an immunofluorescent image analysis method which improved the reproducibility with inter-assay CVs of 2–17%, and had sufficient sensitivity to measure small changes in dystrophin expression in a single DMD patient before and after treatment with an experimental drug^54^. Aeffner et al. further developed IF image analysis as an effective method of analyzing dystrophin expression as a therapeutic biomarker for DMD and BMD^55^. Here, merosin was used as a mask to define the muscle fiber membranes which were independently verified by pathologists.

Despite these improvements several challenges remain. Absolute fluorescence can vary from day to day, and calibration curves are not available^54^. Care needs to be taken with exposure time settings. For example, if the control sample is placed in the middle of the dynamic range, slight changes in expression during therapeutic intervention may be missed^55^. Acquisition factors may also influence the measured fluorescent intensity, negatively affecting comparisons between experiments^56^, and automated IF image analysis requires care in orienting and mounting the tissue to obtain cross-sections with uniform fibers^55^. There is also an urgent need to define “normal” dystrophin expression so that methods can be reproduced by other laboratories as it is apparent that biopsies from healthy individuals used as controls express differing levels of dystrophin^57^. This is further evidenced by the differences in Gd concentration measured across samples from the two healthy subjects without musculoskeletal disease in this study.

Many promising therapeutics are targeting other members of the DGC such as sarcospan, with results showing that an increase in sarcospan in the muscle fiber of the *mdx* mouse model improved sarcolemmal defects^58^. Interestingly, the IF images of sarcospan show that the increased expression was not confined to the sarcolemma. This increase in the sarcoplasm would be identified as a positive signal using k-means clustering, however, would not be measured with a masking approach. The high-resolution images shown in Figs. 1b and 3a show that the amount of dystrophin differs from fiber to fiber. This was consistent with the findings of Beekman et al.^54^, and highlights the necessity for quantitative methods that allow greater data interrogation.

We have shown iMSI is a viable quantitative imaging method for analyzing protein expression in muscle fibers that may be applied to diseases such as DMD or investigate other focal muscle pathologies. The amount of sample required for iMSI reduces the need for invasive surgical biopsies, which recent analyses have shown cause significant anxiety amongst the patients and the caregivers^59^. iMSI may also be multiplexed as the metal tags are readily distinguished from each other with ICP-MS detection. Bodenmiller and colleagues first demonstrated this potential and established IMC for imaging heterogeneous cancer biomarkers^60^, and have since developed software to improve image resolution and cell segmentation^52,61^, and the analysis of mRNA^62^. Highly multiplexed imaging has progressed to include exploring interactions in the pancreas between immune and endocrine cells^63^, mapping the progression of Type 1 diabetes^64^, and quantitative iMSI identified seven markers of early myochardial ischemia^26^ and the three-dimensional expression of a marker for dopamine in the murine mouse brain^23^.

Quantitative imaging of the expression of a clinically important protein such as dystrophin shows the broad applicability of iMSI. The ordered structure of muscle fibers is amenable to high-level multiplexing of additional targets to provide a panel of biomarkers for in-depth tissue characterization, and the determination of the stoichiometry of these proteins. This may result in new knowledge in muscle biology on the fundamental processes of strength and stability of muscle fibers, and potentially identify new therapeutic targets and pathogenic mechanisms.

The US Food and Drug Administration (FDA) industry guide for bioanalytical method validation^18^ was consulted when validating this method. The document was designed as a guide only, as definitive quantitative^65^ bioassays are validated according to “fit-for-purpose” steps similar to those undertaken in standard analytical protocols^66^. Accordingly, the method had appropriate sensitivity to measure different levels of dystrophin expression, exhibited repeatable high linearity with all analyses of our characterized external standards showing an R^2^ greater than 0.99 with all samples analyzed falling within the calibration range, and importantly the analysis was repeatable over replicate samples. The FDA guidance recommended that ligand binding assays should have a CV ± 20%, and the values obtained here for the WT murine and healthy human images were below this value. In general, ligand binding assays such as ELISAs or Western blots are performed on homogenized tissue, reducing the variability inherent in the tissue (e.g. muscle fiber shape, size, etc.). The values obtained here highlight the quantitative repeatability of iMSI as an appropriate, fit-for-purpose method for analyzing dystrophin as a biomarker of therapeutic efficacy in DMD.

## Methods

### Materials

The dystrophin antibody (Mandys8) was purchased from Santa Cruz Biotechnology (Dallas, Texas, USA) and was conjugated with the Maxpar Gd158 reagent by Fluidigm (South San Francisco, CA, USA) who characterized the degree of conjugation and reported a metal atom/antibody ratio of 107.01. Bloxall, mouse on mouse (M.O.M.) basic kit, Vectastain Universal Elite ABC Kit (Anti-Mouse IgG/Rabbit IgG), and ImmPACT DAB peroxidase substrate were purchased from Vector Laboratories (Burlingame, CA, USA), Superblock from Thermo Fisher Scientific (Waltham, MA, USA) and 10× TBS from Bio-Rad (Hercules, CA, USA). 0.1% TBST was made from TBS and Tween-20.

Gadolinium(III) nitrate hexahydrate, Tris–HCl (pH7.4), ethylenediaminetetraacetic acid (EDTA; 10 mM), Polyethylene glycol (Mn 400) and Gelatine from bovine skin (100 mg; Type B) were purchased from Sigma Aldrich (Castle Hill NSW, Australia).

Grace Bio-Labs (Bend, OR) supplied 6 Hybriwell gasket (20 × 9.8 mm) and clear polycarbonate cover with two ports (item number 612107, depth 0.25 mm, volume 50 µL). Ultrapure HNO_3_ and Gd standard were supplied by Choice Analytical (Thornleigh, New South Wales, Australia).

### Mouse models

Wildtype (C57BL/6J) and *mdx* (C57Bl/10ScSn background) mouse quadriceps tissues were harvested from mice maintained following guidelines established by the Institutional Animal Care and Use Committee at the University of California, Los Angeles, and approval for the mice in this study was granted by the UCLA Institutional Animals Care and Use Committee (IACUC) (#2000-029-61D). Muscles were frozen in OCT, sectioned at 10 µm thickness, and stored at − 80 °C until use.

### Human tissue

Human muscle biopsies were obtained with informed consent from healthy individuals, patients, or, for minor patients, from their parents/guardians/legally-authorized representatives by the UCLA Center for Duchenne Muscular Dystrophy (UCLA CDMD) under the University of California Los Angeles IRB‐approved protocol (#11‐001087) and all methods were performed in accordance with required guidelines and regulations. Skeletal muscle biopsies from the vastus lateralis were embedded in OCT, frozen in liquid nitrogen, and stored at − 80 °C. Two samples were obtained from normal individuals without a history of musculoskeletal disease (Healthy 1, female, age 80 and Healthy 2, male, age 18). The DMD samples in this study were selected from four DMD subjects with different dystrophin mutations, dystrophin expression by immunofluorescence, and clinical presentations.

### Histological preparation

After air-drying, the mouse muscle cryosections were washed with TBS before incubation with M.O.M. blocking reagent (Vector Labs, Burlingame, CA) for 60 min. Samples were further washed with TBST before a 5 min incubation with M.O.M. diluent followed by a 30 min incubation with the gadolinium-conjugated primary anti-dystrophin antibody (Mandys-8; 1:100 concentration optimized via dilution). The slides were then washed with TBST, rinsed with double distilled H_2_O, and allowed to air dry overnight.

The human muscle biopsy cryosections were air dried, washed with TBS and incubated with Bloxall blocking reagent (Vector Labs, Burlingame, CA) for 10 min. The samples were then washed with TBST before a 30 min incubation with gadolinium-conjugated primary anti-dystrophin antibody (Mandys-8; 1:500 concentration optimized via dilution). The slides were washed with PBS, rinsed with double distilled H_2_O, and allowed to air dry overnight. The samples that were labeled with the avidin–biotin secondary followed a similar protocol. After the primary incubation and PBS washes, the slide was incubated with the biotinylated secondary antibody for 30 min, washed in PBS, and then incubated for 30 min with the ABC reagent. The biotinylated secondary antibody and the ABC reagent were prepared according to the kit instructions. The slides were then washed in PBS before the DAB peroxidase substrate was applied until a strong color was observed.

For immunofluorescence, sections were incubated in primary antibody in PBS-3% BSA at 4 °C overnight with dystrophin Rod domains (NCL-DYS2, 1:50, Leica Biosystems) and Laminin (L9393, 1:25, Sigma-Aldrich) after pre-incubation with 3% BSA in PBS for 30 min. Primary antibodies were detected by the secondary antibodies FITC donkey anti-mouse visualized dystrophin (715-095-150, Jackson immunology, 1:300) and Texas red anti-rabbit for laminin (711-076-152, Jackson immunology, 1:300). All sections were mounted in Hardset Vectashield-dapi (Vector Laboratories) and visualized using an Axioplan 2 fluorescence microscope with Axiovision 3.0 software (Carl Zeiss Inc).

### Preparation of IMSI standards

Matrix matched gelatine standards were prepared according to a previously described and validated method^27^. A stock solution of 25,000 µg L^−1^ Gd was prepared by dissolving 323.89 mg of gadolinium(III) nitrate hexahydrate in 100 uL pH 7.4 aqueous buffer comprising 100 mM Tris–HCl, 10 mM EDTA, and 1% w/w polyethylene glycol. A series of gelatine standards were prepared by dilutions of this stock solution in the buffer to levels shown in Table 2 and addition of 100 mg of gelatine to 900 µL of the dilutions at 53 °C with periodic vortexing.

**Table 2:** Concentrations of gelatine standards (concentrations given in ng g^−1^).

Element	Blank	1	2	3	4	5
Gd	1.33 ± 0.06	16.78 ± 0.15	61.15 ± 0.40	241.08 ± 1.86	892.85 ± 4.87	3523.06 ± 13.53

Flat homogeneous standard sections suitable for laser ablation were prepared by adhesion of 6 Hybriwell gaskets and clear polycarbonate covers with two ports to a glass slide. The slide was heated to 53 °C for 1 min on a dry heat block before pipetting 50 µL of the metal-gelatine standard mixture via the port.

The standard slide was cooled to room temperature for 30 min and then to − 20 °C in a freezer for 30 min or until the gel was frozen. The adhesive gasket and polycarbonate covers were then removed, and the standards stored at room temperature until required for use.

To determine the concentration of the standards, 100 µg of each standard was dissolved in 1 mL of HNO_3_, diluted to 5 mL, and analysed by solution ICP-MS. Rhodium was used as an inline internal standard. An Agilent Technologies 7700x series ICP-MS (Agilent Technologies, Mulgrave, Vic, Australia) was used with sample introduction via a micromist concentric nebulizer (Glass Expansion, West Melbourne, Vic, Australia) and a Scott type double pass spray chamber cooled to 2 °C. ICP-MS extraction lens parameters were selected to maximize the sensitivity of a 1% HNO_3_:HCl solution containing 1 ng mL^−1^ of Li, Co, Y, Ce, and Tl. Helium was added into the octopole reaction cell to reduce interferences. Calibration curves were constructed and processed using Agilent Technologies Masshunter 4.3 (version C.01.03) software.

### Mass spectrometry imaging

All mass spectrometry imaging experiments were performed on a New Wave Research NWR-193 excimer laser (Kennelec Scientific, Mitcham, Victoria, Australia) hyphenated to an Agilent Technologies 7700x series ICP-MS (Agilent Technologies, Mulgrave, Victoria, Australia). To maximize sensitivity and ensure a low oxide formation (ThO/Th < 0.3%) with LA-conditions, a NIST 612 Trace Element in Glass CRM was ablated. High purity liquid Ar boil-off was used (Ace Cryogenics, Castle Hill, New South Wales, Australia) as the carrier gas. The low resolution image was obtained with a laser spot size of 15 µm and a scan speed of 60 µm s^−1^ at 20 Hz. The high resolution images were reconstructed from two orthogonal ablation passes using a super resolution reconstruction technique^28^. Briefly, the first pass consisted of the ablation of a 300 µm × 300 µm region of interest with a unidirectional scan with a spot size of 15 µm and a scan speed of 30 µm s^−1^ at 20 Hz. In the second pass the same 300 µm × 300 µm region of interest was ablated in an orthogonal direction and the line scans offset by 7.5 µm from the first pass.

### Image processing

Image processing was performed using MATLAB for reconstructing the high resolution images, followed by FIJI for image filtering. The MATLAB code was written in-house and is available from (https://github.com/Elemental-Bio-Imaging-Facility). The default FIJI Gaussian filter and the DeconovolutionLab2^67^ plugin for Richardson-Lucy total variance deconvolution (RLTV) were used in this experiment. Processing was performed on both samples and calibration standards.

Median, Otsu’s, Sauvola’s and Phansalkar’s methods were implemented with in-house MATLAB code. The median method finds the median value of the image and excludes all the lower values from further calculations. Otsu’s method searches the intensity histogram of the image for the middle value between two regions of equivalent variance within the intensity range (i.e. the center point in the range of low frequency intensities between two peaks of high frequency intensity) and then excludes the data lower than this value. Both Sauvola’s and Phansalkar’s methods first apply a mean filter to the image and then use this mean filtered image to find the standard deviation within a search area (i.e. kernel) to attribute positive and negative signal using different search parameters detailed in their respective publications^29,32^. K-mean and k-median were implemented by a python wrapper (https://github.com/djdt/ckwrap) used to implement the Ckmeans.1d.dp algorithm and find the optimal k-mean and k-median clustering results^68^.

### Statistical analysis

The averages per section obtained from segmentation were put into Microsoft Excel to calculate the concentration and CV for each sample.

## Supplementary Information

Supplementary Information.
