# Supplementary material for: Quantitative immuno-mass spectrometry imaging of skeletal muscle dystrophin
Source: Sci Rep. 2021 Jan 13;11:1128. doi: 10.1038/s41598-020-80495-8 (PMC7806610; doi:10.1038/s41598-020-80495-8)
Supplement: Supplementary file 1 — Supplementary Information. [file 41598_2020_80495_MOESM1_ESM.docx]

Supplementary

Quantitative immuno-mass spectrometry imaging of skeletal muscle dystrophin

David P. Bishop, Mika T. Westerhausen, Florian Barthelemy, Thomas Lockwood, Nerida Cole, Elizabeth Gibbs, Rachelle H. Crosbie, Stanley F. Nelson, M. Carrie Miceli, Philip A. Doble, Jonathan Wanagat


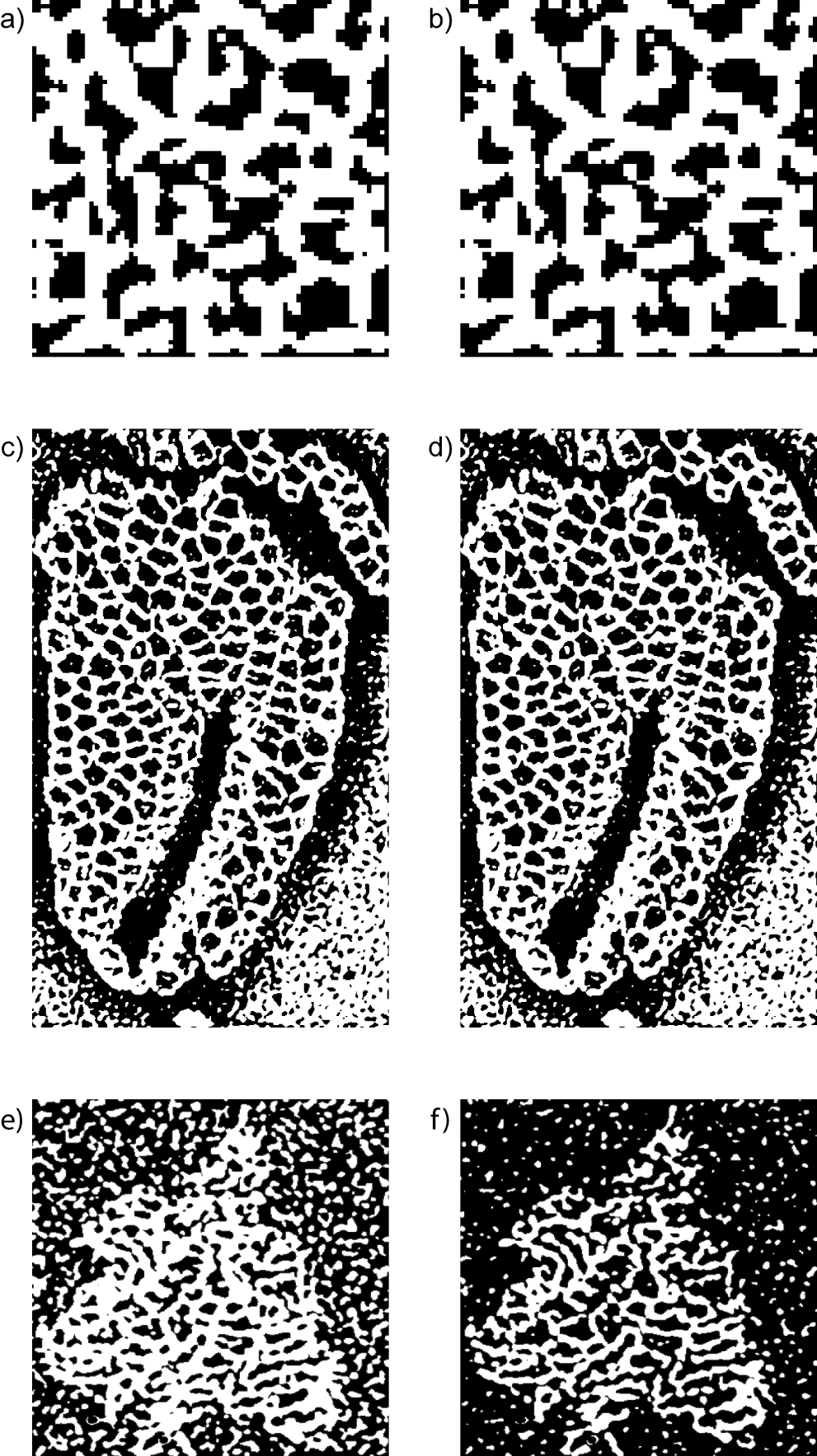


Supplementary Figure 1. Sauvola (a) and Phansalkar (b) segmentation masks on wild-type murine quadriceps 300 µm x 300 µm; Sauvola (c) and Phansalkar (d) segmentation masks on an entire biopsy of Healthy 2; k-medians (e) and k-means (f) segmentation masks on DMD 4.

Supplementary Table 1. Gd concentrations obtained via iMSI as a proxy for dystrophin expression for each of the segmentation methods. Concentrations expressed as µg kg^-1^ Gd ± CV

| Sample | Raw | Median | Otsu’s | Sauvola | Phansalkar | k-medians | k-means |
| --- | --- | --- | --- | --- | --- | --- | --- |
| WT mouse^a^ | 426 ± 7% | 639 ± 6% | 763 ± 8% | 545 ± 7% | 427 ± 7% | 650 ± 7% | 736 ± 10%^b^ |
| *mdx* mouse^a^ | 55.3 ± 28% | 78.4 ± 22% | 88.7 ± 20% | 71.2 ± 19% | 56.9 ± 26% | 77.2 ± 22% | 85.0 ± 21% |
| Healthy 1^a^ | 121 ± 33% | 195 ± 24%^b^ | 365 ± 19%^b^ | 258 ± 23%^b^ | 163 ± 27%^b^ | 313 ± 33% | 382 ± 19%^b^ |
| Healthy 2^a^ | 97.3 ± 26% | 183 ± 21% | 275 ± 19%^b^ | 187 ± 20%^b^ | 108 ± 19%^b^ | 236 ± 9%^b^ | 286 ± 17% |
| DMD 1^c^ | 18.9 | 45.6 | 116 | 57.0 | 47.0 | 68.9 | 99.2 |
| DMD 2^c^ | 36.9 | 59.4 | 84.7 | 65.5 | 41.5 | 66.1 | 86.5 |
| DMD 3^c^ | 29.5 | 40.3 | 46.5 | 39.5 | 29.5 | 40.4 | 48.4 |
| DMD 4^c^ | 32.6 | 53.7 | 98.9 | 52.4 | 33.8 | 69.4 | 100 |

^a^n=7 consecutive muscle sections

^b^outlier removed after Grubb’s test (g_crit_=1.938 for n=7).

^c^single sections were analyzed from these samples so CV is not applicable (na)
